# Tropical Data: Approach And Methodology As Applied To Trachoma Prevalence Surveys

**DOI:** 10.1080/09286586.2023.2249546

**Published:** 2023-12-12

**Authors:** Emma M Harding-Esch, Clara R Burgert-Brucker, Cristina Jimenez, Ana Bakhtiari, Rebecca Willis, Michael Dejene Bejiga, Caleb Mpyet, Jeremiah Ngondi, Sarah Boyd, Mariamo Abdala, Amza Abdou, Yilikal Adamu, Addisu Alemayehu, Wondu Alemayehu, Tawfik Al-Khatib, Sue-Chen Apadinuwe, Naomie Awaca, Marcel S Awoussi, Gilbert Baayendag, Dieng Badiane Mouctar, Robin L Bailey, Wilfrid Batcho, Zulficar Bay, Assumpta Bella, Nassirou Beido, Yak Yak Bol, Clarisse Bougouma, Christopher J Brady, Victor Bucumi, Robert Butcher, Risiate Cakacaka, Anaseini Cama, Mamoudou Camara, Eunice Cassama, Shorai Grace Chaora, Amel Chenaoui Chebbi, Alvin Blessings Chisambi, Brian Chu, Abdulai Conteh, Sidi Mohamed Coulibaly, Paul Courtright, Abdi Dalmar, Tran Minh Dat, Thully Davids, Mohamed El Amine DJAKER, Maria de Fátima Costa Lopes, Djore Dézoumbé, Sarity Dodson, Philip Downs, Stephanie Eckman, Bilghis Elkhair Elshafie, Mourad Elmezoghi, Ange Aba Elvis, Paul Emerson, Emilienne EE Epée, Daniel Faktaufon, Mawo Fall, Aréty Fassinou, Fiona Fleming, Rebecca Flueckiger, Koizan Kadjo Gamael, Mackline Garae, Jambi Garap, Katie Gass, Genet Gebru, Michael M Gichangi, Emanuele Giorgi, André Goépogui, Daniela Vaz Ferreira Gómez, Diana Paola Gómez Forero, Emily W Gower, Anna Harte, Rob Henry, Harvy Alberto Honorio-Morales, Dunera R Ilako, Amadou Alfa Bio Issifou, Ellen Jones, George Kabona, Martin Kabore, Boubacar Kadri, Khumbo Kalua, Sarjo Kebba Kanyi, Shambel Kebede, Fikreab Kebede, Jeremy D Keenan, Amir B Kello, Asad Aslam Khan, Houria KHELIFI, Janvier Kilangalanga, Sung Hye KIM, Robert Ko, Susan Lewallen, Thomas Lietman, Makoy Samuel Yibi Logora, Yuri A Lopez, Chad MacArthur, Colin Macleod, Felix Makangila, Brehima Mariko, Diana L Martin, Michael Masika, Patrick Massae, Marilia Massangaie, Hadley S Matendechero, Tsedeke Mathewos, Siobhain McCullagh, Aboulaye Meite, Elsa Palma Mendes, Hirpa M Abdi, Hollman Miller, Abdellahi Minnih, Sailesh Kumar Mishra, Tuduetso Molefi, Aryc Mosher, Nerkoua M’Po, Francis Mugume, Robson Mukwiza, Consity Mwale, Stephen Mwatha, Upendo Mwingira, Scott D Nash, Christophe NASSA, Nebiyu Negussu, Cece Nieba, Jean Claude Noah Noah, Christian O Nwosu, Nicholas Olobio, Rapheal Opon, Alexandre Pavluck, Isaac Phiri, Merelesita Rainima-Qaniuci, Kristen K Renneker, Martha Idalí Saboyá-Díaz, Fatoumata Sakho, Salimato Sanha, Virginia Sarah, Boubacar Sarr, Celia L Szwarcwald, Ahmad Shah Salam, Shekhar Sharma, Fikre Seife, Gloria Marina Serrano Chavez, Mactar Sissoko, Henis Mior Sitoe, Oliver Sokana, Fentahun Tadesse, Fasiah Taleo, Sandra Liliana Talero, Youcef Tarfani, Amsayaw Tefera, Rabebe Tekeraoi, Andeberhan Tesfazion, Abubaker Traina, Lamine Traoré, Julián Trujillo-Trujillo, Edridah M Tukahebwa, Praveen Vashist, Ernest B Wanyama, Supriya D.p. WARUSAVITHANA, Titus K Watitu, Sheila West, Ye Win, Geordie Woods, Aya YAJIMA, Georges Yaya, Alem Zecarias, Solomon Zewengiel, Akoi Zoumanigui, Pamela J Hooper, Tom Millar, Lisa Rotondo, Anthony W Solomon

**Affiliations:** London School of Hygiene & Tropical Medicine, UK; RTI International/LSHTM, USA; Sightsavers, UK; ITI, USA; ITI, USA; Sightsavers/Independent, Ethiopia; Sightsavers/ University of Jos, Nigeria; RTI International, UK; ITI, USA; Ministry of Health, Mozambique; Programme National de Santé Oculaire, Niger; Addis Ababa University, Ethiopia; RTI International, Ethiopia; Fred Hollows Foundation, Ethiopia; Ministry of Health, Yemen; Ministry of Health and Medical Services, Nauru; Ministère de la Santé Publique, Democratic Republic of Congo; Ministère de la Santé et de L’Hygiène Publique, Togo; Ministry of Health, Uganda; Ministère de la Santé, Senegal; London School of Hygiene & Tropical Medicine, UK; Ministère de la Santé, Benin; RTI International, Mozambique; National Programme for Blindness Control/MoPH, Cameroon; Programme Nationale de Santé Oculaire, Niger; Ministry of Health, South Sudan; Programme national de lutte contre les maladies tropicales négligées (PNMTN), Burkina Faso; University of Vermont, USA; National Integrated Programme for the Control of Neglected Tropical Diseases and Blindness (PNIMTNC), Burundi; London School of Hygiene & Tropical Medicine, UK; MOHMS/WHO, Fiji; Fred Hollows Foundation, New Zealand; Ministère de la Santé, Guinea; Ministério de Saúde Publica, Guinea Bissau; National Statistical Agency, Zimbabwe; Hedi Raeïs Institute of Ophthalmology, Tunisia; Lions Sight First Eye Hospital, Malawi; International Trachoma Initiative, USA; Ministry of Health and Sanitation, Sierra Leone; Organisation pour la Prévention de la Cécité, Mali; Division of Ophthalmology, University of Cape Town, Cape Town, South Africa, South Africa; Ministry of Human Development and Public Services, Somalia; Ministry of Health, Vietnam; Ministry of Health and Social Services, Namibia; World Health Organization, Algeria; Ministry of Health, Brazil; Ministère de la Santé Publique, Tchad; Fred Hollows Foundation, Australia; RTI International/ Sightsavers, USA; RTI International, USA; Sudan National Trachoma Control Programme, Sudan; Libyan National prevention of blindness, Libya; Programme National de la Santé Oculaire et de la lutte contre l’Onchocercose, Côte d’Ivoire; International Trachoma Initiative, USA; Department of Disease Control, Sub direction of NTDs, Cameroon; Ministry of Health and Medical Services, Fiji; RTI International, Mozambique; Ministère de la Santé, Benin; SCI Foundation, UK; RTI International/ LSHTM, USA; Ministère de la Santé et de l’Hygiène Publique, Cote d’Ivoire; Ministry of Health, Vanuatu; Port Moresby General Hospital, Papua New Guinea; Task Force for Global Health, USA; Federal Ministry of Health, Ethiopia; Ministry of Health, Kenya; Lancaster University, UK; Ministère de la Santé Publique, Guinea; Secretaria de vigilância em saúde - Ministério da saúde, Brazil; Ministerio de Salud y Protección Social, Colombia; University of North Carolina at Chapel Hill, USA; London School of Hygiene & Tropical Medicine, UK; U.S. Agency for International Development, USA; Ministry of Health of Peru, Peru; University of Nairobi (UON), Kenya; Ministry of Health, Benin; Sightsavers, UK; Ministry of Health and Social Welfare, Tanzania; Programme national de lutte contre les maladies tropicales négligées (PNMTN), Burkina Faso; Programme Nationale de Santé Oculaire, Niger; Blantyre Institute for Community Outreach, Malawi; Ministry of Health and Social Welfare, Gambia; Federal Ministry of Health, Ethiopia; Federal Ministry of Health, Ethiopia; University of California, San Francisco, USA; World Health Organization, Congo; Ministry of National Health Services, Pakistan; World Health Organization, Algeria; Saint Joseph Hospital, DRC; World Health Organization, Philippines; Port Moresby General Hospital, Papua New Guinea; Division of Ophthalmology, University of Cape Town, Cape Town, South Africa, South Africa; University of California, San Francisco, USA; Ministry of Health, South Sudan; SACAICET / MINISTERIO DEL PODER POPULAR PARA LA SALUD, Venezuela; MacArthur/Tapert Global Health, USA; London School of Hygiene & Tropical Medicine, UK; Ministry of Public Health, DRC; Direction Régionale de la Santé de Kayes, Mali; Centers for Disease Control and Prevention, USA; Ministry of Health, Malawi; Kilimanjaro Christian Medical Centre, Tanzania; Ministério da Saúde, Mozambique; Ministry of Health, Kenya; Federal Ministry of Health, Ethiopia; Sightsavers, UK; Ministère de la Santé et de l’Hygiène Publique, Cote d’Ivoire; Ministry of Health Angola, Angola; Oromia Regional Health Bureau, Ethiopia; Secretaria de Salud del Vaupés, Colombia; Ministère de la santé, Mauritania; Nepal Netra Jyoti Sangh, Nepal; Ministry of Health and Wellness, Botswana; U.S. Agency for International Development, USA; Ministère de la santé, Benin; Ministry of Health, Uganda; Ministry of Health and Child Care, Zimbabwe; Ministry of Health, Zambia; Ministry of Health, Kenya; Ministry of Health, Tanzania; The Carter Center, USA; Ministère de la santé et de l’hygiène publiques, Burkina Faso; Federal Ministry of Health, Ethiopia; Ministère de la Santé et de l’Hygiene Publique, Guinea; National program for preventing blindness/independent., Cameroon; Sightsavers, Nigeria; Federal Ministry of Health, Nigeria; Ministry of Health, Uganda; RTI International/ Sightsavers, USA; Ministry of Health and Child Care, Zimbabwe; World Health Organization, FIji; International Trachoma Initiative, USA; Pan American Health Organization (PAHO), USA; Ministry of Health, Guinea; Ministério de Saúde Publica, Guinea Bissau; Fred Hollows Foundation, UK; Ministère de la Santé, Senegal; Oswaldo Cruz Foundation, Brazil; Ministry of Public Health, Afghanistan; Nepal Netra Jyoti Sangh, Nepal; Federal Minsitry of Health, Ethiopia; Ministerio de Salud Pública y Asistencia Social de Guatemala, Guatemala; Ministère de la Santé, Senegal; Ministry of Health, Mozambique; Solomon Islands Ministry of Health and Medical Services, Solomon Islands; Federal Ministry of Health, Ethiopia; Vanuatu Ministry of Health, Vanuatu; Escuela Superior de Oftalmología, Instituto Barraquer de América, Colombia; Ministère de la Santé, Algeria; Federal Ministry of Health, Ethiopia; Ministry of Health and Medical Services, Kiribati; Ministry of Health, Eritrea; Ministry of Health, Libya; Ministère de la Santé, Mali; Ministerio de Salud y Protección Social, Colombia; Ministry of Health, Uganda; All India Institute of Medical Sciences, India; Ministry of Health, Kenya; World Health Organization, Egypt; Ministry of Health, Kenya; Johns Hopkins University, USA; Myanmar MOH, Myanmar; Sightsavers, USA; World Health Organization, Philippines; Ministère de la Santé et de la Population, CAR; Ministry of Health, Eritrea; Ministry of Health, Eritrea; Ministère de la Santé et de l’Hygiene Publique, Guinea; International Trachoma Initiative, USA; Sightsavers, UK; RTI International, USA; World Health Organization, Switzerland

**Keywords:** Trachoma, *Chlamydia trachomatis*, Tropical Data, survey, prevalence

## Abstract

**Purpose:**

Population-based prevalence surveys are essential for decision-making on interventions to achieve trachoma elimination as a public health problem. This paper outlines the methodologies of Tropical Data, which supports work to undertake those surveys.

**Methods:**

Tropical Data is a consortium of partners that supports health ministries worldwide to conduct globally standardised prevalence surveys that conform to World Health Organization recommendations. Founding principles are health ministry ownership, partnership and collaboration, and quality assurance and quality control at every step of the survey process. Support covers survey planning, survey design, training, electronic data collection and fieldwork, and data management, analysis and dissemination. Methods are adapted to meet local context and needs. Customisations, operational research and integration of other diseases into routine trachoma surveys have also been supported.

**Results:**

Between 29^th^ February 2016 and 24^th^ April 2023, 3373 trachoma surveys across 50 countries have been supported, resulting in 10,818,502 people being examined for trachoma.

**Conclusion:**

This health ministry-led, standardised approach, with support from the start to the end of the survey process, has helped all trachoma elimination stakeholders to know where interventions are needed, where interventions can be stopped, and when elimination as a public health problem has been achieved. Flexibility to meet specific country contexts, adaptation to changes in global guidance and adjustments in response to user feedback have facilitated innovation in evidence-based methodologies, and supported health ministries to strive for global disease control targets.

## INTRODUCTION

Trachoma, caused by conjunctival infection with the bacterium *Chlamydia trachomatis*, is the leading infectious cause of blindness worldwide and is targeted for global elimination as a public health problem by the year 2030. ^1^ Ocular infection with *C. trachomatis* causes a follicular conjunctivitis, which can include the specific sign “trachomatous inflammation—follicular” (TF). ^2^ Repeated infections^3^ over many years result in progressive conjunctival scarring in the upper eyelid; in some individuals, after multiple repeat infections, ^4^ that scarring may cause the eyelid margin to turn inwards such that the eyelashes touch the eyeball. This is known as “trachomatous trichiasis” (TT). ^2^ Trachoma elimination is defined as: a prevalence of TT “unknown to the health system” of <0.2% in adults aged ≥15 years, in each formerly-endemic district; a prevalence of TF in children aged 1–9 years <5%, sustained for at least two years in the absence of ongoing antibiotic mass drug administration (MDA; a single oral dose of azithromycin or 1% tetracycline eye ointment twice daily for 6 weeks^5^), in each formerly-endemic district; and the existence of a system to identify and manage incident TT cases, using defined strategies, with evidence of appropriate financial resources to implement those strategies. ^6^

**Figure 1.**
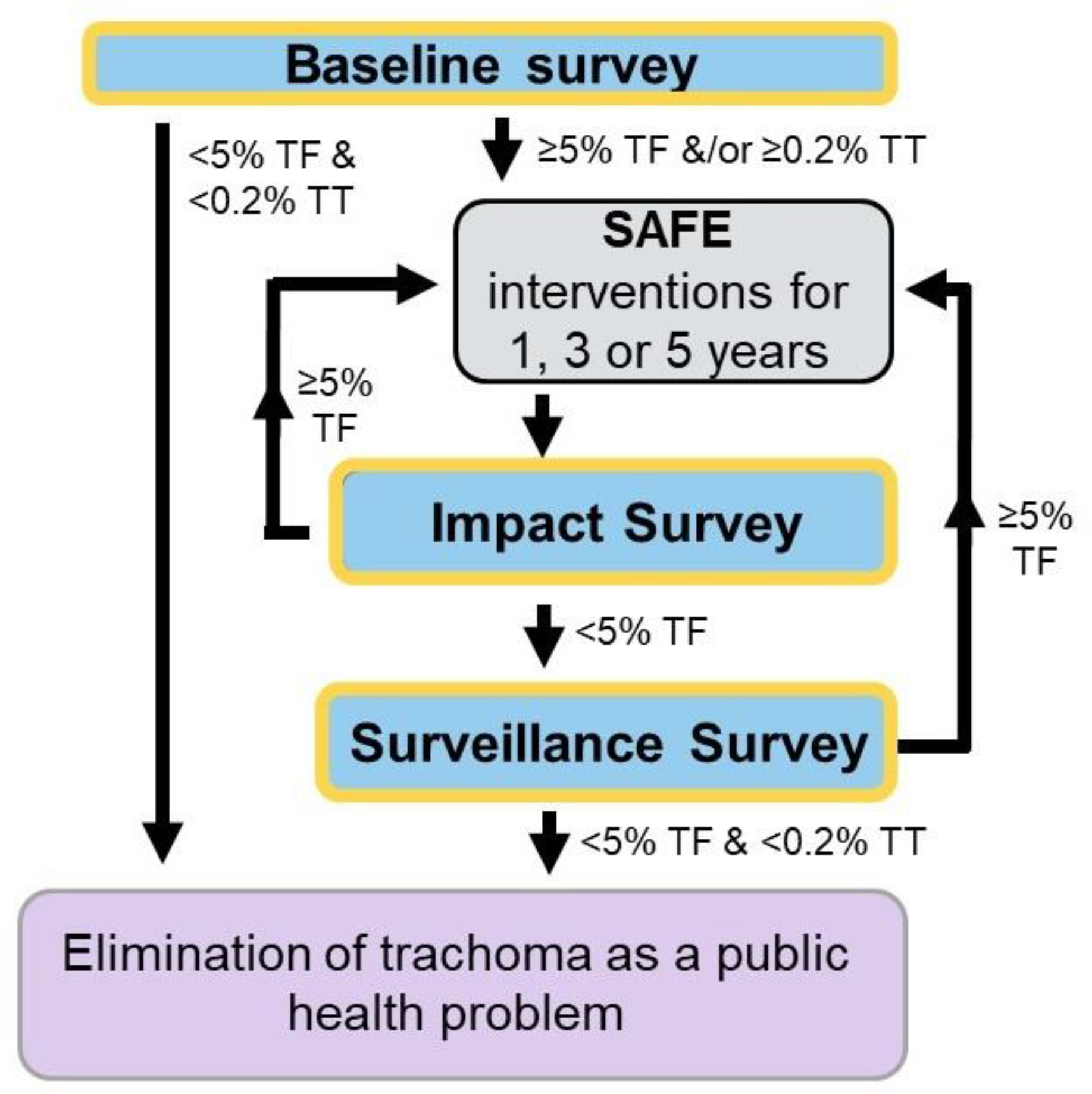
Decision-making for baseline, impact and surveillance surveys and SAFE (surgery, antibiotics, facial cleanliness and environmental improvement) interventions for trachoma elimination^8, 9^ TF: trachomatous inflammation—follicular; TT: trachomatous trichiasis

The World Health Organization (WHO)-endorsed strategy to achieve elimination is known as SAFE: **S**urgery for TT, **A**ntibiotics to clear infection, and **F**acial cleanliness and **E**nvironmental improvement to limit transmission. ^7^ Planning for implementation of the A, F and E components of the SAFE strategy is undertaken based on the prevalence of TF in children aged 1–9 years (TF1–9) at the level of the “evaluation unit” (EU), where EUs are generally “the normal administrative unit for health care management, consisting of a population unit between 100,000–250,000 persons”. ^8^ (This often corresponds to a “district” or similar second-level administrative division.) The following recommendations are used (Figure 1): ^8,9^ offer at least five years of A, F and E where TF ≥30% in 1–9-year-olds, three years of A, F and E where TF1–9 is 10–29.9%, and one year of A, F and E when TF1–9 is 5–9.9%. Similarly, public health level-actions to deal with TT are guided by its prevalence in ≥15-year-olds: if the <0.2% elimination threshold is not met, public health-level TT surgery services, including active case finding and outreach services, are recommended; if the threshold is met, routine eye health care services should be provided to manage any remaining (plus incident) TT cases. ^10,11^

Baseline surveys establish the pre-intervention prevalence of trachoma in the EU, with the prevalence category determining the number of years of A, F, and E required. Impact surveys are conducted 6–12 months after the last planned round of MDA is completed, to determine if further interventions are needed. Surveillance surveys are undertaken at least 24 months after an impact survey shows that TF1–9 is <5%, to determine if interventions against TF need to be re-introduced. If the prevalence of TF1–9 has been maintained below the elimination threshold in the absence of ongoing MDA at surveillance survey, no further surveys are required in that EU. ^12^ Baseline, impact and surveillance surveys aim to estimate the prevalence of both TF1–9 and TT in ≥15 year-olds, but sample sizes are generally calculated based on parameters relating to TF and may not provide reliable TT prevalence estimates. TT-only surveys are recommended for determining the prevalence of TT in individuals aged ≥15 years in certain epidemiological contexts; ^13^ they attempt to balance moderate precision with acceptable cost.

Demonstrating that TF and TT prevalence thresholds have been reached through well-conducted surveillance surveys in previously trachoma-endemic EUs are key elements of a country’s dossier when submitting their request to WHO for validation of national elimination of trachoma as a public health problem. ^6^ Population-based trachoma prevalence surveys are, therefore, essential for both decision-making on SAFE interventions and validating an elimination claim. ^14,15^

From 2012–2016, the Global Trachoma Mapping Project (GTMP) used standardised methods and approaches to support health ministries to complete baseline mapping of all accessible, suspected trachoma-endemic districts worldwide. ^16,17^ The data collected with GTMP support were instrumental in determining where SAFE implementation was and was not needed to eliminate trachoma as a public health problem. ^17,18^ Through its focus on health ministry ownership and close collaboration between governments and partners, facilitating survey coordination and planning and national intervention planning following results availability, GTMP helped strengthen international commitment to global trachoma elimination. ^15,19,20^ For example, the data resulted in a significant increase in the volume of the antibiotic azithromycin (Zithromax^®^) donated by Pfizer through the International Trachoma Initiative, ^21^ from approximately 50 million doses annually in the years prior to the GTMP to an average of approximately 100 million doses annually for several years afterwards.

In 2016, “Tropical Data” (www.tropicaldata.org) was launched to further the work of the GTMP. ^22^ While the GTMP was funded to support completion of baseline surveys, Tropical Data was established to support all trachoma survey types (baseline, impact, surveillance, TT-only) ^23^ and, where requested, prevalence survey needs for other neglected tropical diseases (NTDs). ^24^ Here we present Tropical Data’s approach and methodology for the different types of trachoma survey.

## MATERIALS AND METHODS

**Figure 2.**
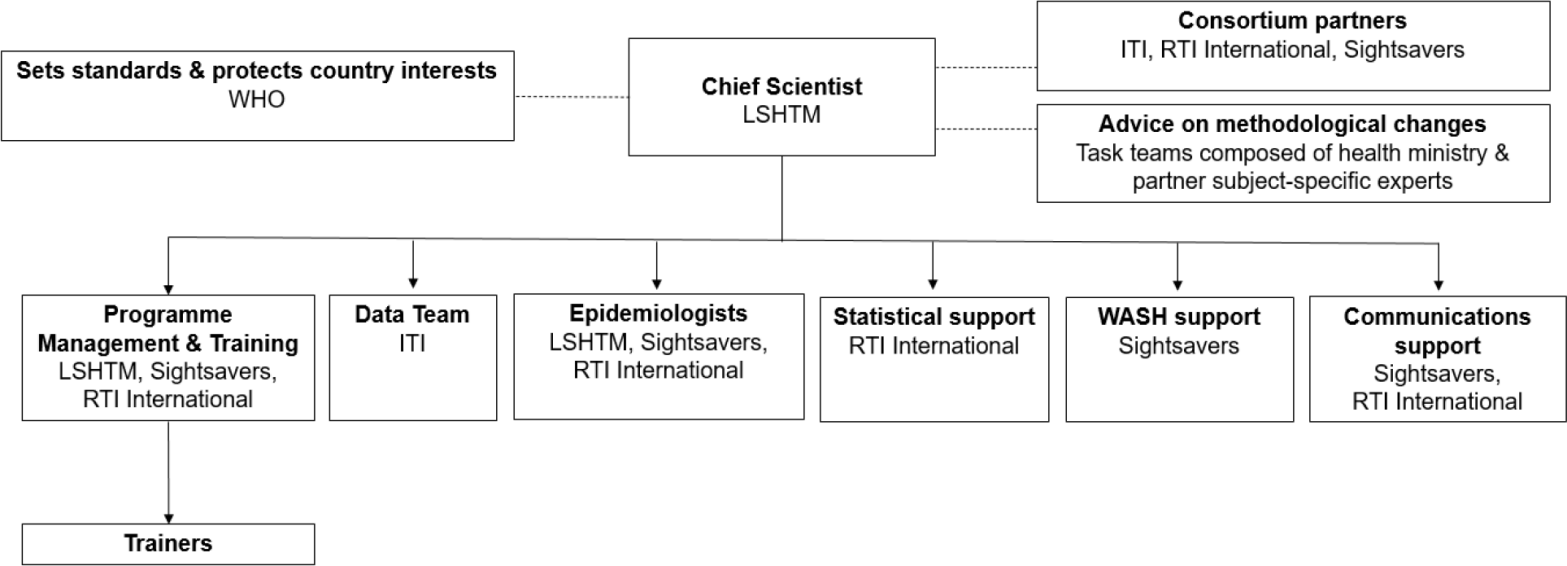
Tropical Data Organisation Chart for supporting trachoma surveys ITI: International Trachoma Initiative; LSHTM: London School of Hygiene & Tropical Medicine; WHO: World Health Organization; WASH: Water, Sanitation and Hygiene.

Tropical Data supports national programmes to conduct globally standardised, epidemiologically robust prevalence surveys. We are a core team of partners that includes ITI, RTI International, Sightsavers, and the London School of Hygiene & Tropical Medicine (LSHTM) (Figure 2). WHO sets the standards we conform to and protects country interests, while specific task teams composed of subject-specific experts advise us on methodological changes to implement. The Tropical Data team consists of one full-time Chief Scientist; one full-time data analyst; one full-time systems analyst; one full-time programme manager; five part-time epidemiologists; more than 1800 certified fieldworkers and supervisors; and more than 350 certified trainers. Ad hoc statistics; water, sanitation and hygiene (WASH); and communications support is also available. Support for academic publications is provided by the epidemiologists and data team. Although the service provided is free for any country to use, countries are responsible for funding the fieldwork components themselves, with support from implementing partners in many countries.

### Principles

The core Tropical Data team developed a set of principles (https://tropicaldata.knowledgeowl.com/help/tropical-data-principles) that are adhered to for all the surveys that Tropical Data supports. The principles are:

1. We support rigorous standardisation, quality assurance and quality control throughout the survey process, to ensure data meaningfully inform programmatic decision-making and are of sufficient quality for inclusion in dossiers submitted to WHO to support disease control, elimination, or eradication claims.
2. The health ministry owns the process and data.
3. We collaborate as equals, foster inter- and intra-country collaboration, encourage similar partnership between governments, donors, non-governmental organisations (NGOs) and other implementing partners, WHO, and academic institutions.

### 1. Quality and standardisation

**Figure 3.**
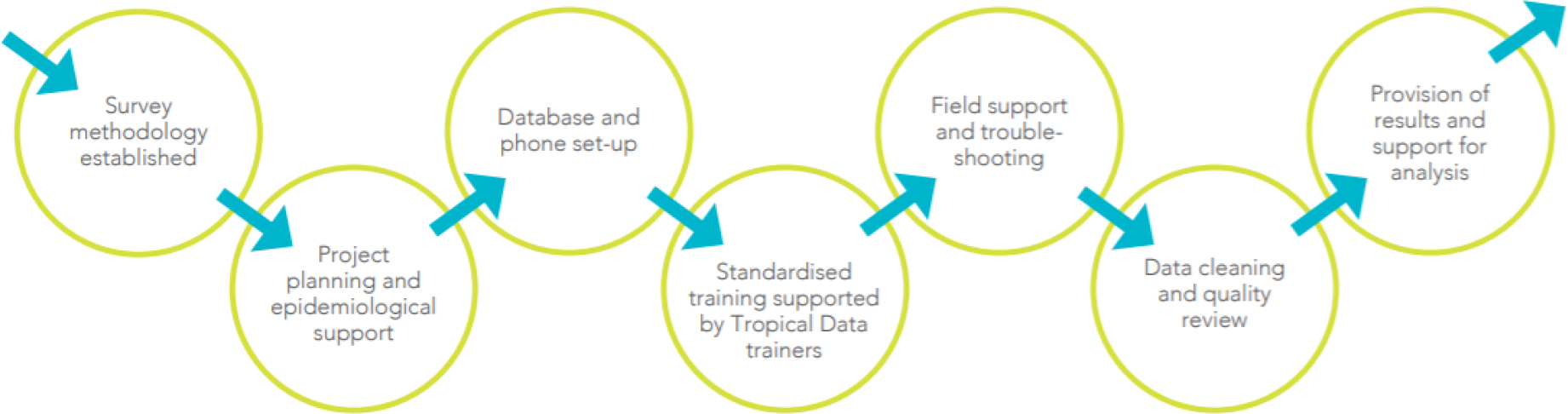
Tropical Data Quality and Standardisation Process

Figure 3 outlines the Tropical Data quality and standardisation process. The process starts with the use of established survey methods and subsequently includes planning, training, field support, data cleaning, and provision of results.

#### Quality and standardisation: Planning and Support

We provide a range of templates, tools, and information on a central resource website (https://tropicaldata.knowledgeowl.com/help/) to support health ministries as they plan a survey. The process starts when health ministries contact us, either via email or by submitting a formal application on the online submission portal (http://application.tropicaldata.org/). We use this system to manage, coordinate, and sign off on project requests. We follow the same criteria as used by the GTMP for where to conduct baseline surveys, including the EU being likely to be trachoma-endemic on the basis of existing data. ^25^ Impact surveys can take place at least 6 months after the last planned MDA round has been completed, and surveillance surveys 2 years after the most recent impact survey returned a TF prevalence <5%. For any survey type, fieldwork is not recommended if field teams will be put at risk due to insecurity.

An epidemiologist is assigned to support the health ministry with protocol drafting or finalisation and, if required, with cluster sampling. The epidemiologist advises on survey planning and preparations more broadly, alongside the Tropical Data programme manager and the data team (including data collection forms and EU identification codes). The programme manager additionally ensures training requirements are met, including the availability of Tropical Data-certified trainers, and supports the procurement of all required specialised materials; we are able to provide smartphones for data collection, and magnifying loupes, 3D glasses^26^ and follicle size guides^27^ to assist with trachoma grading.

#### Quality and standardisation: Survey Design

Scientific overview starts during the planning stage, with the review of survey protocols by dedicated subject-specific epidemiologists, and WHO sign-off that the protocol conforms to WHO recommendations. To support protocol development in-line with WHO recommendations, ^13, 28^ we created a protocol writing guide (https://tropicaldata.knowledgeowl.com/help/protocol-writing-guide). In brief, the WHO-recommended population-based prevalence survey methodology for trachoma uses two-stage cluster sampling in each EU. ^28^ The primary sampling units (clusters) are selected with probability proportional to population size. ^8^ Within each cluster, for the second sampling stage, households are selected by simple random sampling and systematic sampling when there is a complete list of households at the cluster level, and compact segment sampling when there is not. ^29,30^

Surveys are conducted at EU level, which informs programmatic decisions for intervention delivery. Therefore, if a normal administrative division for health care is split into two or more EUs due to large population size, it is vital to ensure that programmatic decisions can be implemented at the EU level, including if TF1–9 prevalence category results turn out to be different. If this would not be possible, WHO agreement for surveying large (>250,000) population size EUs is sought. For multiple smaller districts (<100,000) to be combined into one EU, they should be socio-economically similar, geographically contiguous, and the combination should make sense programmatically and geo-politically. If these criteria cannot be met, surveying smaller EUs is recommended.

Table 1 outlines the design parameters and survey sample size inputs (for the single population proportion for precision formula^31^) for baseline, impact, surveillance, and TT-only surveys. Baseline surveys may be powered to detect a TF prevalence of either 10% (±3%) or 4% (±2%), depending on the expected TF prevalence (perhaps based on historical data, current prevalence in neighbouring districts, and/or geographical, socio-economic and political context). ^28^ An expected prevalence of 10% (±3%) is often appropriate because EUs with TF1–9 ≥10% qualify for three annual MDA rounds, compared with one round for those with a prevalence 5–9.9%.^8, 9^ The 4% (±2%) expected prevalence is used for determining whether the EU TF1–9 <5% elimination threshold has been reached, indicating that trachoma is not a public health problem, as recommended at the 3rd Global Scientific Meeting on Trachoma in 2010. ^9, 28^ When the population of the EU is small, the finite population correction should be applied. ^32,33^

The number of clusters needed per evaluation unit is determined by dividing the number of individuals to enumerate by ([number of households per cluster]x[mean number of individuals of target age group per household]). If the number of clusters calculated is <30, we ask health ministries if they would like us to check the demographic data from previous surveys in the specific EUs so that we can be more confident that the sample size will be met, as the calculation then uses more precise data rather than (often national) estimates. To have confidence in the TT prevalence estimates, we can also increase the number of clusters to 30, but generally not to >30, as statistical modelling has demonstrated that acceptable precision around the prevalence estimate is obtained if 30 clusters are surveyed, even if the sample size is not met. ^13^ A key consideration is cluster accessibility, and we ask health ministries to inform us at the protocol development stage if they are aware of any potential issues, such as insecurity or flooding, so we can assist with mitigation in the field. Cluster replacement is not recommended, as it can affect the geographical representativeness of the survey.

We encourage health ministries to target a fixed number of households rather than a fixed number of individuals, to avoid data collection errors in the field, such as rushing fieldwork or convenience sampling. If a cluster has fewer than 20 households, we recommend that teams report this to their supervisor, who will notify the national programme and Tropical Data. Tropical Data and the national programme will then discuss options for cluster completion. The sampling of clusters within the EU (minimum 20, maximum 30^28^), and sampling of households within the cluster (usually 20–30 households, depending on how many a field team can comfortably survey in a day), are undertaken to adhere to the principles of equal probability random sampling, or its best approximation given the circumstances. ^28^

#### Quality and standardisation: Training

We have adapted the GTMP’s training system, ^35^ adding new material and refinements as a result of field teams’ feedback and experience, the latest research, and changes in relevant guidelines and recommendations (see supplementary material). We offer two distinct standardised training packages for trachoma, one that supports baseline, impact and surveillance surveys, ^36^ and the other for TT-only surveys. ^37^ Materials are available in English, French, Spanish, Portuguese, and Arabic.

**Figure 4.**
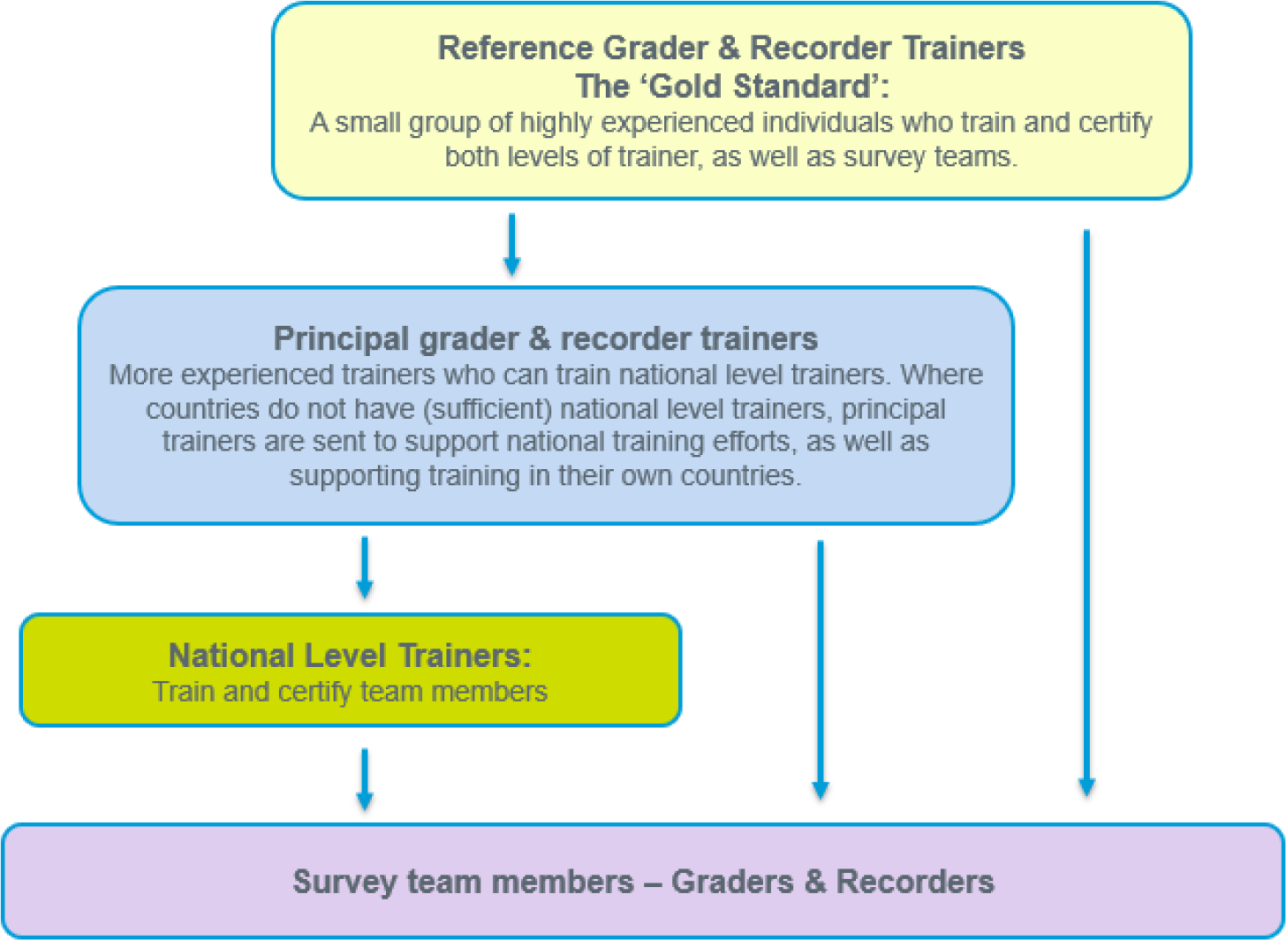
Tropical Data Trachoma Training Cascade

There are two levels of training: training of trainer (TOT) workshops and national-level trainings. We organise TOTs directly, and usually run them at a global or regional level to train principal and/or national-level trainers. Principal trainers can train and certify national-level trainers and survey team members (Figure 4). Where a country has a large trachoma programme and requires larger numbers of teams, TOT workshops are organised at a national level. In addition to confirming familiarity and competence with the Tropical Data survey methods and technical skills, TOT workshops focus on training practices to impart this knowledge to others.

National-level trainings are typically organised by the countries themselves, and we support them by ensuring that certified trainers have been identified and that guidelines are followed. Candidates selected to be national-level trainers or part of the survey teams are usually chosen by the health ministries, with guidance on selection criteria provided in the relevant Tropical Data training manual.^36,37^ National-level trainers can only certify survey team members, not further trainers (Figure 4). We aim to re-certify national-level trainers if they have not been active in the field and/or trained in the past two years. For previously trained survey team graders and recorders who have not been active for over 6 months, refresher training should be undertaken.

Graders and recorders are trained separately at the beginning of the trachoma baseline, impact, and surveillance survey training system. A grader’s role is to examine community residents for signs of trachoma. Trainee graders must successfully complete the initial grader qualifying workshop, which includes two “Inter-Grader Agreement” (IGA) assessments, with trainees required to obtain a kappa of ≥0.7 in the diagnosis of TF in both. The first is a classroom-based IGA using 50 photographs, where the TF grade has been unanimously agreed by five international-expert graders. ^16^ The second is a field-based IGA examining 50 children, of whom a minimum of five (but no more than 35) must have TF, ^36^ with the grade determined by the grader trainer. If there are fewer than 15 cases, the IGA becomes harder to pass. ^16^ Also, as TF prevalence decreases, the proportion of “borderline” compared to “clear” cases increases. ^38,39^ Consequently, in some instances, trainees must travel further in-country^40^ or to other countries^41^ in order to be trained. On the rare occasions where this is not possible, we have used photo-based training followed by enhanced supervision during fieldwork. ^40^ Follicle size guides ^27^ have been incorporated into the training (as well as for fieldwork) to improve accuracy of TF diagnosis. A recorder’s role is to capture data from eye examinations as well as from a household questionnaire (assessing WASH access) in an Android smartphone using the Tropical Data application. Recorders are certified using a recorder reliability test which consists of trainees entering correct data from fictitious households in the Tropical Data app; a score of ≥90% is required to be certified. Successful trainees progress to the team training, which lasts two days and brings the grader and recorder roles together to look in-depth at key aspects of survey methodology, including sampling, household selection, obtaining consent, and supervision. It involves both theoretical teaching and discussion, as well as classroom-based roleplay. One of the final parts of the team training sees teams practise working together in the field.

Training for TT-only surveys is structured differently due to differences in survey requirements and the rarity of TT cases, which makes field IGAs unfeasible. Given that only individuals aged ≥15 years are surveyed and no WASH or TF data are collected, the survey process moves more quickly. Consequently, the grader can also act as the recorder and is trained to carry out both roles. Graders selected to participate in TT-only surveys are expected to have previously successfully completed the training for baseline, impact, and surveillance surveys. To qualify as grader-recorders for TT-only surveys, trainees must pass an Objective Structured Clinical Examination (OSCE), which includes assessing whether the trainee follows the correct sequence for examination, including trichiasis health management questions; can correctly identify different trichiasis diagnoses using images (3D, or 2D if 3D glasses unavailable; for each trichiasis diagnosis there are two images, one with the eye looking straight, one with the eye looking up); can correctly identify trachomatous scarring (TS) from five photographs; and can accurately enter all the data into the Android smartphone. ^37^ Each step within each competency is marked as “meets expectation” or “below expectation”; trainees with no more than three grades of “below expectation” may proceed to field practice. 3D glasses have been incorporated into TT-only training to increase the specificity of trichiasis diagnosis. ^26^

#### Quality and standardisation: Data collection and fieldwork

Data are captured on Android smartphones using the Tropical Data application, a customised version of Open Data Kit. The application is freely available in the Google Play Store (https://play.google.com/store/apps/details?id=com.nafundi.tropicaldata.collect.android&hl=en_US&gl=US). We use electronic data capture, eliminating the need for paper forms. Electronic records are preferable because they are backed up in electronic systems, are collected and reported from a single source and do not need transcription (where additional errors can be introduced), include data validations to minimise missing data and improve quality through range and consistency checks, and allow for rapid review and feedback with field teams. ^16,42^ Our current survey forms are available on the Tropical Data resource pages (https://tropicaldata.knowledgeowl.com/help) in English, French, Hindi, Portuguese, Spanish, Swahili and Vietnamese.

To support data quality, globally-standardised survey forms written in Extensible Markup Language (XML) are coded with built-in data validation and quality control. Collected data are stored on a MySQL server using the Amazon RDS service. The server is located in the us-east-1 region and data are encrypted at rest. Collected data are accessible from a central password-protected Ruby on Rails web application hosted on Heroku. All connections to the service use Transport Layer Security (TLS) 1.2 encryption; the certificates are managed by Amazon and Heroku.

For trachoma baseline, impact and surveillance surveys, a household questionnaire is administered after household head consent, which collects global positioning system coordinates and WASH data. WASH variables are consistent with the WHO/UNICEF Joint Monitoring Programme for Water Supply, Sanitation and Hygiene (JMP) core questions for households. ^43^ Graders then examine both eyes of each consenting individual using binocular magnifying loupes (×2.5) and adequate lighting. Follicle size guides, which we routinely provide, should be used for the diagnosis of TF. ^27^ All consenting residents aged one year or above from each selected household are examined for the clinical signs of trichiasis, TF and TI (trachomatous inflammation—intense). Since our last methods update in 2019, the presence or absence of trichiasis has been recorded for the upper and lower eyelids separately, following the changed definition of TT agreed upon at the 4th Global Scientific Meeting on Trachoma (GSM4), which excludes trichiasis affecting the lower eyelid only. ^44^ Individuals with trichiasis are also examined for TS; if the eyelid cannot be everted, we assume that TS is present in the analysis because eyelids that are heavily scarred can be difficult to evert. Health management questions regarding trichiasis (upper or lower eyelid) are also asked, using the local definition of a health worker who can offer management. By having the application automatically prompt to ask these follow-up questions, the recorder checks and confirms the TT diagnosis, increasing data accuracy for this rare phenotype. ^12^ We also ask that graders verify the health management responses by paying attention to the presence or absence of a trichiasis surgical scar. Individuals who respond that they have not received an offer of surgery or epilation, or respond “Don’t know” to the questions, are considered “unknown to the health system”. Patient treatment (antibiotic treatment for active trachoma) and management (referral to a health facility for TT) follows each country’s national guidelines.

For TT-only surveys, no WASH data are collected. The grader-recorder examines each person aged ≥15 years for the clinical signs of trichiasis (upper and lower eyelid separately), and the same processes as for baseline, impact and surveillance surveys (diagnosis of TS, health management questions, evidence of surgical scar, referral to health facility) are followed.

Teams return to households at the end of each day, if time allows, to examine household members from the target age-group (1–9-year-olds for baseline, impact and surveillance surveys; ≥15-year-olds for TT-only surveys) who were absent at the initial time of examination. Field coordination and supervision are typically done by the health ministry, with support from implementing partners in some countries. Supervisors often act as the link between the data team and survey teams, facilitating communication and resolution of any issues. We strongly recommend that all supervisors are also certified as graders and/or recorders, or at a minimum, attend the team’s training to be fully conversant on the methodologies being used.

#### Quality and standardisation: Data management and analysis

Data are stored on the smartphone until a data-enabled mobile network or WiFi signal is available, at which point they are uploaded and stored in encrypted form on a section of a Cloud-based server dedicated to each project. The data are only accessible to designated health ministry personnel on the password-protected Tropical Data data management site (https://www.tropicaldata.org/projects). As data are uploaded, they are received and available in near-real-time and are regularly reviewed by a dedicated data analyst, enabling consistent, rapid feedback across all study sites to resolve issues while teams are still in the field. To provide support to field teams during data collection, the data analyst sends summary reports along with cleaning questions and recommendations multiple times per week. Data cleaning is conducted by the data analyst; however, as data are owned by the health ministry, data cleaning is only performed at the request of or following approval by the health ministry. Any changes are logged in a table accessible by the health ministry. All raw data are preserved.

Once data collection and data cleaning for an EU are complete, data are analysed in R statistical software using automated analysis pipelines. The code used to analyse the data is available on GitHub (https://github.com/itidat/tropical-data-analysis-public). EU-level means of adjusted cluster-level proportions are calculated, with TF prevalence in 1–9-year-olds adjusted for age in one-year bands, and TT prevalence in ≥15-year-olds adjusted for gender and age in five-year bands, according to the most recent census. ^16^ The 95% confidence intervals (CIs) are calculated by taking the 2.5th and 97.5th centiles from an ordered list of 10,000 bootstrap iterates of the adjusted cluster-level outcome proportions, randomly resampled with replacement. ^45,46^ WASH data are analysed as per JMP categorisations, ^43^ focusing on the number and proportion of surveyed households with an improved drinking water source, a drinking water source within 30 minutes, and an improved latrine.

Following data analysis, the Chief Scientist conducts a “final-for-Tropical Data” data review. The results then circulate through a two-step approval process, in which review and approval are completed by the health ministry, in order to be considered final and made available for download. Designated users can then access the approved results in various EU-level summary formats on a dedicated downloads page on the data management site, including an expanded trichiasis summary aimed to help support national programmes plan their TT management.

To support data sharing and use, health ministries can elect to automatically share the trachoma point prevalence data with the joint ITI/WHO GET2020 database, so that ITI and WHO can help populate applications for donated drugs^47^ and trachoma dossier templates. ^6^ Health ministries can also elect to automatically share trachoma prevalence category data with the open-access Trachoma Atlas (https://www.trachomaatlas.org/) and for countries in WHO’s Africa region, the ESPEN (Expanded Special Project for Elimination of Neglected Tropical Diseases) Portal (https://espen.afro.who.int/). Data sharing is facilitated through an Application Programming Interface (API) to provide data directly for national data servers. One example of this is integration with the national NTD database using District Health Information System II (DHIS2) software.

We encourage international dissemination of the data by each collaborating health ministry, including at conferences and in peer-reviewed publications. ^48–55^ This helps share the survey methodologies used, the estimated prevalence results, and other analyses such as associations with WASH variables. It can also demonstrate trachoma prevalence trajectory over time, which can inform national programme decision-making and operational research. Publication also means data have been externally reviewed by the time they are presented to the WHO-convened dossier review group for consideration of validation of elimination of trachoma as a publication health problem. The Tropical Data core team supports health ministries with the dissemination process on request, and has produced a virtual workshop on writing trachoma survey publications, available on the Tropical Data YouTube channel (https://tropicaldata.knowledgeowl.com/help/publications-workshop).

#### Customisation, Operational Research, and Supporting Other Diseases

We also support survey customisation, operational research, and integration of other diseases into routine trachoma surveys. For all of these, we have maintained our routine processes (logistics and planning, protocol review, training, data collection and management support, routine trachoma survey outcome analyses, and scientific oversight) whilst collaborating closely with partners who support the non-standard components, such as additional training requirements, collection of samples in the field, laboratory testing, and non-standard data analyses. These bespoke surveys necessarily require more resources and time at every stage of the survey process, and we have developed an operational research checklist (https://tropicaldata.knowledgeowl.com/help/or-checklist) to assist planning, expectations and timelines.

The high-quality, highly standardised data that we help collect also represents a resource of huge value for secondary data analyses for research purposes. ^56–58^ Tropical Data’s commitment to country ownership of data requires countries to approve any secondary use of survey data and to take the action that gives data access to any third party. While health ministries have total access to, and control over, their data (including data dictionaries, prevalence estimates and raw individual-level data), health ministry data owners often enlist the support of the Tropical Data data team to prepare individual datasets for download, which health ministries then share with the third-party research team.

#### Ethical considerations

Requirements for ethical clearance for trachoma surveys vary from country to country. The LSHTM observational ethics committee has approved Tropical Data support for surveys (reference number 16105), which adhere to the guidelines of the Declaration of Helsinki. In-country ethical clearance for surveys may or may not be required: in some jurisdictions they are conceptualised as a routine programme monitoring or public health surveillance activity. ^59^ However, obtaining informed consent (in the local language) for examination from each person or parent/guardian for children is required and is the responsibility of the survey team. In some countries, verbal assent from children aged 9–17 years is also required by local authorities. Verbal consent for examination by each examined individual is registered in the Tropical Data smartphone application used for data collection, and countries may also decide to obtain written informed consent using paper forms based on local guidelines. Provisions for treating individuals with active trachoma and referral for cases of unmanaged TT (and other eye conditions where appropriate) are documented in the survey protocols. With the advent of emergencies such as the COVID-19 pandemic, additional ethical considerations to safeguard teams and communities are considered. ^60–62^

### 2. Health ministry ownership of the survey process and data

**Figure 5.**
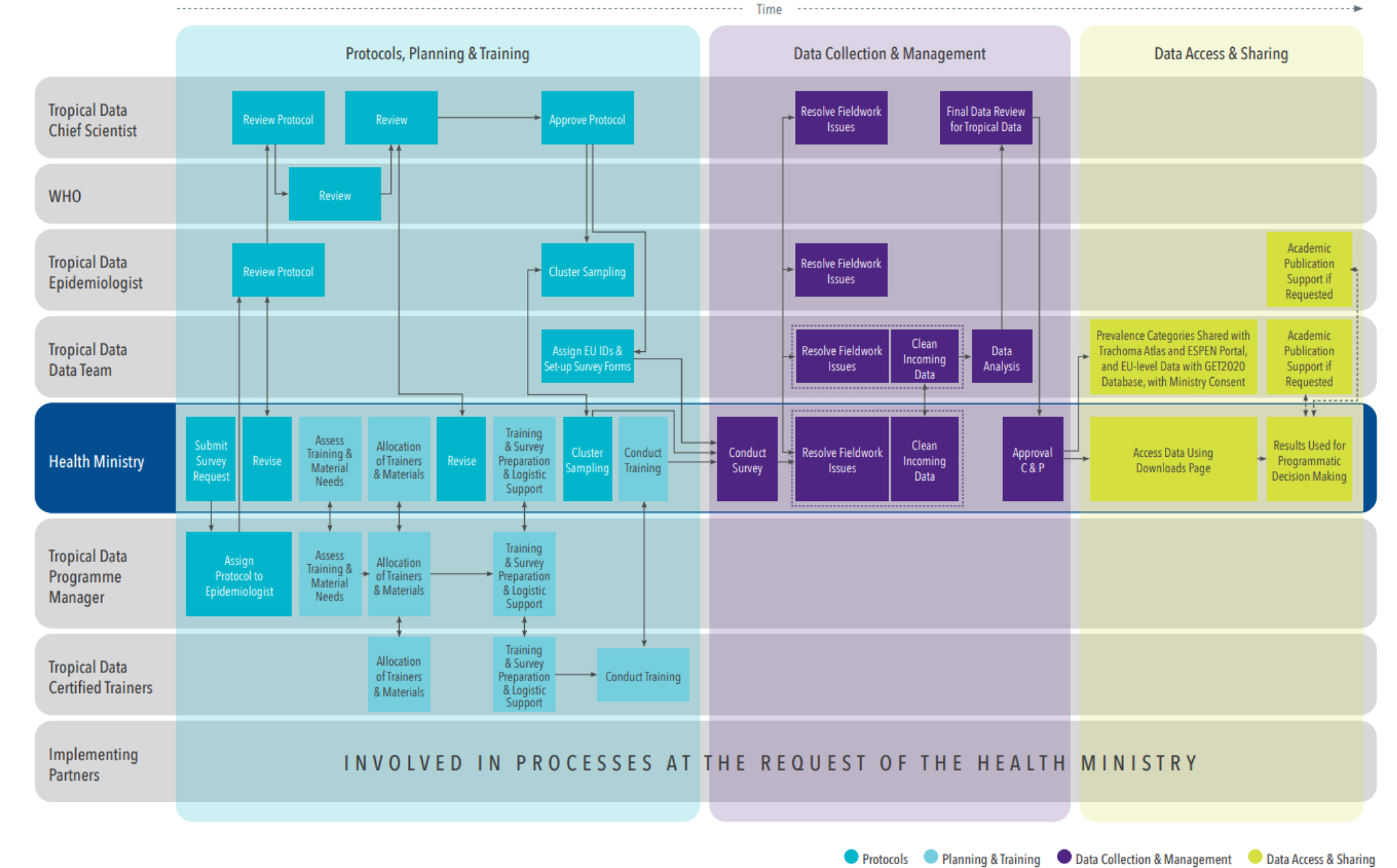
Roles and responsibilities during the Tropical Data survey process EUID: Evaluation Unit Identification number; WHO: World Health Organization

Health ministries lead, and are the central partner in, every stage of the Tropical Data process, and all data collected using Tropical Data tools are owned by the respective health ministry. Figure 5 provides a schematic of the survey process, roles and responsibilities.

### 3. Collaboration and partnership

Our core team is the result of collaboration and partnership between the supporting consortium of partners. The wider Tropical Data team is built from collaboration with governments, donors, NGOs and other implementing partners, WHO, and academic institutions. We work closely with all these partners to support countries throughout the survey process. Our interactions create other opportunities for working together. Our training system provides multiple formal and informal avenues for inter- and intra-country collaboration, with international TOTs offering an opportunity for learning from and supporting each other and sharing of best practices. This process of collaboration and learning is furthered through the global network of trainers and the countries they support, enabling us to continually learn, evaluate, review and adapt our processes. ^63^ We disseminate updates in our biannual newsletter, “The Download” (https://tropicaldata.knowledgeowl.com/help/tropical-data-news), as well as at international conferences, including meetings of the WHO Alliance for GET2020, and through our role as an observer to the International Coalition for Trachoma Control (https://www.trachomacoalition.org/).

## RESULTS

Between Tropical Data’s inception on 29^th^ February 2016 and 24^th^ April 2023, we have supported 3,373 trachoma surveys across 50 countries, including eight of the 17 countries validated by WHO as having eliminated trachoma as a public health problem (Benin, Gambia, Malawi, Mali, Myanmar, Nepal, Togo and Vanuatu). In these surveys, Tropical Data-certified graders examined a total of 10,818,502 residents for signs of trachoma and confirmed that MDA should be started or continued in 951 EUs, was not needed in 449 EUs, and could be stopped in 890 EUs, and helped countries confirm that 695 EUs had reached the elimination threshold.

We have additionally supported survey customisations, operational research, and surveys for diseases other than trachoma. Customisations, where there are additions to routine surveys to inform further programmatic decision-making, include collection of lymphatic filariasis (LF) morbidity questions, inclusion of trachoma MDA participation questions, questions on time since trichiasis surgery, and adaptations to the household WASH questionnaire to meet local needs. ^50^ We have also employed a different sampling methodology for refugee camps (random household selection instead of two-stage cluster sampling) due to the camp structures and the nature of data available. Following assessment of geostatistical modelling to generate more precise TT prevalence estimates in Ethiopia, ^64^ this approach is being further piloted to determine probabilities of EUs meeting elimination criteria. In terms of operational research, we have supported the collection of eye swabs to test for ocular *C. trachomatis* infection and serological testing of dried blood spots. ^65–70^ Other operational research studies have included evaluating the feasibility and accuracy of photography for trachoma grading^71–73^ and options for face shields as protective equipment in trachoma surveys during the COVID-19 pandemic. ^74^ Support for integration of other diseases with trachoma has included soil-transmitted helminths in Peru, ^75^ and more extensive integration of multiple NTDs in Ethiopian refugee camps. ^76^ As part of pilots to explore whether the Tropical Data process and methodologies can be applied to other NTDs, ^77^ we have partnered with the Pan American Health Organization (PAHO) and Unlimit Health (formerly SCI Foundation) to support surveys on LF in Guyana^78–80^ and schistosomiasis in Dominican Republic.

## DISCUSSION

Tropical Data and GTMP form the largest series of infectious disease surveys ever conducted. This has been made possible through having a clear and common goal (trachoma elimination), standardised methodologies, and quality assurance and quality control at every step of the survey process. ^25^ By supporting impact and surveillance surveys in addition to baseline surveys, we not only support health ministries to know where interventions are needed, but also where interventions can be stopped and when elimination has been achieved. By continuing to apply the Tropical Data principles and adapting our processes to meet country needs, we support national programmes to conduct evidence-driven surveys to achieve sustainable development goal 3.3, to “end the epidemics of ... neglected tropical diseases”. ^1^

To do this effectively, we must continually learn and adapt to international recommendations and country requests and needs. Major adaptations we have implemented have been in response to international changes, such as the GSM4 and WHO/UNICEF JMP recommendations made in 2019. At the same time, actions to address both informal feedback from workshops, such as international TOTs, and more formal feedback surveys sent to all Tropical Data users, have been implemented. One major change to the service was made in 2017, when we introduced support for TT-only prevalence surveys for use in particular epidemiological contexts, ^13^ with these being one of the WHO-recommended methods for assessing TT prevalence at GSM4. ^44^ Our continuing work on geostatistical modelling^64^ in collaboration with Lancaster University’s Centre for Health Informatics, Computing and Statistics (CHICAS) WHO Collaborating Centre (WHOCC) on Geostatistical methods for NTD Research will enable us to explore whether another GSM4 recommendation of combining survey data from several neighbouring EUs could provide more precise and cost-effective prevalence estimates, and be incorporated in the Tropical Data platform. Since programmatic decisions for trachoma are made based on the point prevalence estimate, obtaining greater precision around this estimate is important to help determine if further interventions are needed. Furthermore, recommendations from the WHO Informal Consultation on End-Game Challenges for Trachoma Elimination in December 2021 included ongoing development of geostatistical analytical approaches and generation of *C. trachomatis* infection and serological data to inform tailored management. ^81^ Working with partners, including the USA Centers for Disease Control and Prevention (CDC) WHOCC for Trachoma, we are working on adapting our processes to incorporate *C. trachomatis* infection data collection into routine surveys for enhanced epidemiological investigation.

A key challenge, both for initiating or completing surveys, is insecurity. As with GTMP, ^25^ Tropical Data strongly advises that surveys should not be conducted if the activity puts field teams at risk. In 2022, 240 districts across 17 countries were reported by the GET2020 Database as being hard-to-reach, for many reasons including insecurity, resulting in unknown prevalence and intervention needs. ^82^ Where surveys have already started but the EU becomes insecure during field activities, the recommendation from WHO is to wait until the security situation improves so that the remaining clusters can be surveyed; not completing the survey as per the original cluster selection would potentially be problematic from a future dossier point-of-view because prevalence estimates would not be representative of the whole EU, following non-random cluster exclusion. No limit has been set on the duration between starting and completing a survey for an EU. Some countries have successfully implemented strategies to overcome security concerns, ^52, 83^ and a decision-tree methodological approach for determining survey needs in countries with ongoing complex emergencies has been proposed. ^84^ However, as some countries are experiencing long-term security challenges preventing teams from conducting fieldwork, this is an area that requires further work if the 2030 elimination goal is to be met.

Another challenge that we are increasingly facing is the scarcity of TF cases in the field. Though this is very good for previously-affected populations, it limits where field IGA training can take place. As a result, Tropical Data trainees have had to travel from Central African Republic to Ivory Coast, from Burundi to Ethiopia, and from six other Latin American countries to Colombia, in order to be trained. The 2018 francophone TOT took place in a non-francophone country (Ethiopia) following difficulties identifying trachoma cases in the field in Senegal in the previous year. ^41^ This has obvious cost and logistical implications. ^85^ In order to future-proof trachoma grading, we have been actively involved in exploring the use of photography in Tropical Data training, supervision, and field grading. ^72, 73^ With partners, we have developed a trachoma photo database (https://trachomaphotos.tropicaldata.org/) for partners to upload photos and associated data, which will serve to support education and research efforts for trachoma elimination and long-term surveillance.

In terms of opportunities, integrated surveys offer the potential for cost-efficient processes, with greater cross-partner and multi-sectoral collaboration, ^78^ as called for in the NTD road map 2021–2030 and PAHO’s disease elimination initiative. ^1, 86^ Through this collaboration, integrated surveys can help with monitoring and evaluation of indicators that are important to both NTDs and other sectors. For example, 100% access to at least basic WASH services is one of the targets in the 2021–2030 road map, and routine collection of WASH access data in surveys, alongside disease-specific data, could help determine whether this target has been met and facilitate prioritisation of WASH interventions. Integrated surveys offer a particular opportunity for “hard-to-reach” populations. ^87^ Populations may be classified as such for a multitude of reasons, including geographic remoteness and insecurity. Integrated surveys maximise opportunities for health ministries to collect data about multiple diseases at the same time, rendering them more cost-effective. To allow data collection on visual impairment and blindness, we are conducting proof-of-concept studies of integrating the Peek visual acuity app into our processes. ^88,89^ However, further work is needed to refine the most robust, yet logistically feasible, methodologies for conducting integrated surveys. The WHO Diagnostics Technical Advisory Group cross-cutting surveillance subgroup has as part of its remit the brief to determine the minimum requirements of multiplex assays, and to provide guidance on survey design and optimising data use. ^90^ We will take recommendations from this group, in addition to lessons learned and findings from operational research, to tailor survey methodologies so that health ministries can cater to the needs of their populations, and ensure no one is left behind.

The potential for integrated surveys and the need for methodology adjustments to serve particular country contexts, highlight the importance of flexibility even within a standardised approach. However, we always work carefully with health ministries when requests for methodological changes are made because deviation from standardisation increases the capacity requirements to prepare for data collection and management (such as the creation of customised forms and analysis code), increases the risk of data errors (for example, field teams rushing to complete additional tasks in time), and subsequently increases the time from survey initiation to results availability. For requests to include additional questions to routine surveys that do not produce programmatically actionable data and/or are not validated methods, ^91^ we have a free-text “notes” field in the resident form, which health ministries can download and analyse themselves.

The 2021–2030 NTD road map sets updated targets, strategy, and timelines, and highlights the importance of high quality and standardised data for monitoring and evaluating progress towards control, elimination and eradication targets. ^1^ The power of a globally standardised approach has been shown through the scale and data quality achieved through GTMP and Tropical Data. ^92^ This has enabled the need for interventions to be determined and prioritised, and the impact of interventions to be assessed. The platform also allows for flexibility to meet specific country contexts, to adapt to global guidance changes as well as user feedback, to innovate to help drive evidence-based methodologies, and to support prevalence surveys for multiple diseases. Ultimately, the process and results help support health ministries in their achievement of global targets for disease control, elimination or eradication.

## Supplementary Material

Supplementary Material

## Figures and Tables

**Table 1.** Inputs to calculate sample size for baseline, impact, surveillance and TT-only surveys^13, 28^

Variable	Input		
	Baseline ^28^	Baseline (with expectation of low TF prevalence), impact, and surveillance ^28^	TT-only ^13^
Expected prevalence (%)^a^	10	4	0.2
Design effect^34^	3.69	2.63	1.47
Standard deviation for 95% confidence interval	1.96	1.96	1.96
Absolute precision (%)	3	2	0.2
Number to examine	1418	970	2818
Non-response rate inflation factor^b^	1.2	1.2	1.2
Targeted number to enumerate^c^	1701	1164	3382

TF: trachomatous inflammation—follicular; TT: trachomatous trichiasis

a For baseline, impact, and surveillance surveys, this is the expected trachomatous inflammation—follicular prevalence in 1–9-year-olds. For TT-only surveys, this is the expected TT prevalence in ≥15-year-olds.

b An arbitrary inflation factor of 1.2 is used unless previous work enables the use of an actual non-response rate for the particular population and setting.

c For baseline, impact, and surveillance surveys, this is the number of children aged 1–9 years to enumerate. For TT-only surveys, this is the number of adults aged ≥15 years to enumerate.
