# Supplementary material for: Tropical Data: Approach And Methodology As Applied To Trachoma Prevalence Surveys
Source: Ophthalmic Epidemiol. Author manuscript; Available in PMC 2024 Dec 12. (PMC10751062; doi:10.1080/09286586.2023.2249546)
Supplement: Supplementary Material [file NIHMS1925952-supplement-Supplementary_Material.docx]

**Supplementary Material 1. Changes to Tropical Data methodology, 2019. ^36, 37^** These took place following responses to our feedback survey, ^63^ World Health Organization recommendations made as a result of the 4th Global Scientific Meeting on Trachoma held in Geneva in November 2018, ^44^ and contemporary WHO/UNICEF Joint Monitoring Programme (JMP) for Water Supply, Sanitation and Hygiene (WASH) core questions for households. ^43^

| **Survey stage** | **Change** |
| --- | --- |
| Planning | - Updated protocol writing guide - Provision of 3D glasses^26^ and follicle size guides^27^ - Provision of a new aide-memoire outlining the sampling process and how to select households |
| Training | - Updated materials for WASH, covering options and definitions for: water source, time to collect water, where adults defecate, handwashing facility - Updated materials to include examination of trichiasis in the upper and lower eyelid separately, and questions on previous healthcare management if trichiasis is present in either the upper or lower eyelid, separately - Greater emphasis on training for trichiasis and trachomatous scarring (TS) grading, including the use of 3D images for training in diagnosis of trichiasis - Inclusion of guidance on use of follicle size guides for diagnosis of trachomatous inflammation—follicular (TF) - Recorder training starting on day 1 - Formal recorder reliability test - New module where recorders demonstrate smartphones to graders - More team training, particularly of trachoma survey methodology - Increased training on sampling and household selection - Updated materials and increased emphasis on supervision |
| Field data collection | - Routine use of follicle size guides for TF diagnosis - Examination and recording of trichiasis separately for upper and lower eyelids - TS and healthcare management questions asked separately for upper and lower eyelid where trichiasis is present - Looking for evidence of surgical scar to confirm response to health management questions and to assist in obtaining a more accurate figure of trichiasis unknown to the health system - Updates to the household WASH questions, including some new responses to choose from - New paper and phone-based supervisor checklists |
| Data management, analysis and reporting | - New data collection forms:   - WASH: new options for water source and latrine types, change in coding for time to get water, no observation for shared latrine, handwashing station question asked regardless of whether or not the household has a latrine   - Eye exam: presence or absence of trichiasis, and questions on previous healthcare management if trichiasis is present, collected for upper and lower eyelid separately. Presence or absence of TS collected if either upper or lower eyelid trichiasis is present - New variables in the data tables (the number of variables doubled for the trichiasis and trichiasis management questions) - Updated analysis code:   - TT defined as trichiasis in the upper eyelid only.   - TS data no longer analysed and no longer included in the definition of TT.   - WASH analysis changes: Delivered water, piped water to neighbour, water kiosk, and packaged water are new “improved” water source options. Container based sanitation is a new “improved” latrine option. Flush/pour flush to open drains is now classified as “unimproved” latrine - Updated data outputs (downloads):   - District Report:     - No longer includes figures that include TS data     - WASH figures that previously mentioned distance now only reference time, to match the data collection forms   - Expanded Trichiasis Report:     - No longer includes figures that include TS data     - No longer includes figures for lower eyelid trichiasis   - WASH Summary:     - WASH figures that previously mentioned distance now only reference time, to match the data collection forms     - Report on presence of handwashing station for all households |
